# Relative Average Look Duration and its Association with Neurophysiological Activity in Young Children with Autism Spectrum Disorder

**DOI:** 10.1038/s41598-020-57902-1

**Published:** 2020-02-05

**Authors:** Dmitry Yu. Isaev, Samantha Major, Michael Murias, Kimberly L. H. Carpenter, David Carlson, Guillermo Sapiro, Geraldine Dawson

**Affiliations:** 1 Department of Biomedical Engineering, Duke University, Durham, NC 27708 USA; 2 Duke Center for Autism and Brain Development and Duke Institute for Brain Sciences, Duke University, Durham, NC 27708 USA; 3 Medical Social Sciences, Northwestern University, Chicago, IL 60622 USA; 4 Department of Civil and Environmental Engineering and Department of Biostatistics and Bioinformatics, Duke University, Durham, NC 27708 USA; 5 Department of Electrical and Computer Engineering, Duke University, Durham, NC 27708 USA; 6 Department of Computer Science, and Department of Mathematics, Duke University, Durham, NC 27708 USA; 7 Department of Psychiatry and Behavioral Sciences, Duke University, Durham, NC 27708 USA

**Keywords:** Diagnostic markers, Autism spectrum disorders

## Abstract

Autism Spectrum Disorder (ASD) is characterized by early attentional differences that often precede the hallmark symptoms of social communication impairments. Development of novel measures of attentional behaviors may lead to earlier identification of children at risk for ASD. In this work, we first introduce a behavioral measure, *Relative Average Look Duration (RALD*), indicating attentional preference to different stimuli, such as social versus nonsocial stimuli; and then study its association with neurophysiological activity. We show that (1) ASD and typically developing (TD) children differ in both (absolute) Average Look Duration *(ALD)* and *RALD* to stimuli during an EEG experiment, with the most pronounced differences in looking at social stimuli; and (2) associations between looking behaviors and neurophysiological activity, as measured by EEG, are different for children with ASD versus TD. Even when ASD children show attentional engagement to social content, our results suggest that their underlying brain activity is different than TD children. This study therefore introduces a new measure of social/nonsocial attentional preference in ASD and demonstrates the value of incorporating attentional variables measured simultaneously with EEG into the analysis pipeline.

## Introduction

Autism spectrum disorder (ASD) is characterized by early attentional differences that often precede the hallmark symptoms of social communication impairments and restricted and repetitive behaviors^1–3^. Attentional processes such as orienting, disengagement from and sustaining attention to relevant stimuli^2,4–6^, and the ability to share attention^7,8^ are foundational for the development of social abilities and social communication. Research has demonstrated deficits in all of these domains of attention in infants and children with ASD^8^. As such, screening and diagnosis place particular emphasis on these behaviors; and early interventions target these attentional processes to facilitate the acquisition of social and communication skills^9–11^. In this work we investigate the associations between attention and simultaneously recorded neurophysiological signals in children with ASD. Our results suggest that even when ASD children show attentional engagement to social content, their underlying brain activity is different than typically developing (TD) children.

A distinctive sign of ASD is robust differences in the amount of attention directed toward social versus nonsocial stimuli, documented across the lifespan and reported as early as 6 months of age in infants who later develop ASD^12–15^. Although neurophysiological recordings (e.g., Event-Related Potentials [ERP]^16^ and spontaneous electroencephalogram [EEG]) and looking behavior paradigms (e.g., via habituation^17^ and gaze^18^, which are often measured with eye-tracking technology^19^, but can also use standard computer vision^20^) have been widely used in autism research, few studies have reported reliable and robust results that combine these measures and jointly analyze them. Our study aims to fill in this gap, investigating the associations between looking/attentional behavior and neurophysiological patterns, registered simultaneously in a synchronized fashion.

In infant studies, a common way to assess social attention is via diverse “habituation” paradigms^17,21–23^. For example, in some studies, static stimuli (faces or objects, i.e., social and nonsocial images) are presented to the participants. Then, look durations, defined as time between initial look at the stimulus and look away, are recorded. From such measures of look duration, various statistics can be derived, including time to habituate (formally, decline attention), peak look duration, mean look duration, and/or number of looks^21,22,24^. Webb *et al*.^21^ showed that toddlers with more severe ASD symptoms based on the Autism Diagnostic Observation Schedule [ADOS]^25^) took significantly longer to habituate to faces than objects (houses). Further, the children with severe ASD took significantly longer to habituate to faces than groups of TD toddlers, as well as toddlers with less severe ASD, developmental delay (DD), siblings of ASD children, and siblings of TD children. In another study^22^, infants who later developed ASD showed shorter look durations to faces than objects, and their peak time to look to faces happened later than in infants who did not develop ASD, suggesting that infants at risk for ASD attend to faces differently than TD infants.

A common method/technology used to measure and analyze looking behavior (or gaze) is eye-tracking^19^. A broad body of literature, including some of the above mentioned papers, has offered insights to visual social attention using this method (see Guillon *et al*.^26^ for a review). Eye-tracking studies are commonly used to assess patterns of attention to dynamic stimuli^6,14^, e.g., movies or changing images, where both social (i.e., people) and nonsocial cues (e.g., toys) are presented and compared. The dynamic nature of the stimuli used in many of these studies is more ecologically valid than studies with static stimuli. In a study of toddlers with ASD, Chawarska *et al*.^6^ showed that the difference between total looking time at faces/objects becomes apparent only when the child was viewing child-directed speech and the actress was making eye contact, referred to as a dyadic bid. In such a scene, the ASD group showed diminished attention to the face and the mouth while their attention to toys in the same scene was increased. This suggests that ASD toddlers have difficulties holding attention to the face particularly in dyadic bid situations. The same experiment, when conducted with 6-month old infants at risk for ASD^15^, demonstrated reduced attention by infants who later developed ASD to the overall social scene, as well as to a person and her face, without any significant difference by types of activities shown (dyadic bids, joint attention, moving toys).

While eye-tracking and habituation studies provide us with rich data on attentional behavior patterns to social/nonsocial stimuli, it is also of interest to understand underlying neurophysiological activity during attention that might help explain differences in social attention. For example, using an ERP paradigm, Dawson *et al*.^27^ showed that TD young children, as well as ASD children receiving an early developmental intervention (Early Start Denver Model^9^), showed increased activity in the theta band and decreased activity in the alpha band while attending to static faces, while ASD children who received treatment as usual showed the opposite pattern (greater activity in theta and less in alpha in response to static toy stimuli). While an extensive body of literature exists for ERP^16^ biomarkers of social attention in ASD^28,29^, fewer studies have focused on measures of spontaneous EEG and their relationship to attention to social dynamic stimuli. In an experiment with TD infants and preschool children that used dynamic stimuli (child-directed speech, manipulating toys, and visual attention to bubbles), theta band power was found to increase during emotionally stimulating conditions (child-directed speech and manipulating toys) as compared to a baseline (bubbles stimulus), supporting previous evidence of a relationship between theta power and attentional states^30,31^. In another study with dynamic stimuli^32^, an EEG connectivity analysis of the data from infants watching social/nonsocial videos was performed, however, the actual type of stimuli was not a factor influencing the EEG results.

Given the potential role of attention in influencing underlying neurophysiological activity, it is of interest to simultaneously measure both attention and EEG and jointly consider them for analysis. This was done in several EEG studies in which the participant’s behavior was videotaped synchronously with EEG recording^32–35^, and subsequently looking behavior (looks at the screen), as well as motor behavior (significant motion), and/or emotional behavior (crying, excessive smiling) was coded offline. However, in those studies, the coded participant’s behavior was used only for data preprocessing^33–35^, or group comparisons of the behavioral variables^32^, and joint analysis of EEG and behavior was not explicitly performed. To the best of our knowledge, in the area of ASD research, only one study so far performed simultaneous eye-tracking (as a proxy to attention) and EEG analysis in an experiment with joint attention in a small sample of high-functioning autistic children^36^. The authors reported a positive correlation between cumulative fixation duration on face and beta and gamma band relative power. Our study uses a different age range and introduces significant additional analysis both of the attentional signal (as a measure of *relative* responses to social vs. nonsocial stimuli) and of the EEG signal, as well as their interaction.

In the present study, we explore a method to jointly study EEG and synchronized looking behavior during the same experiment. We introduced a new measure*, Relative Average Look Duration* (*RALD*), a normalized measure indicating attention preference when comparing different stimuli, i.e., social vs. nonsocial, allowing a within-subject comparison of relative attention directed to different stimuli. This allows for an individual measure of *attentional preference*. Furthermore, young children with autism are not compliant with instructions to sit quietly without any video stimuli while spontaneous EEG are collected. Thus, it is considered standard in the field of autism research not to include a video-free baseline but rather to examine how the brain responds to different types of stimuli during spontaneous EEG recording^37^; the proposed *RALD* naturally addresses this challenge. As mentioned above^4,21^, previous studies have shown differences in preference to social as compared to nonsocial stimuli in children with ASD. Here we develop a new measurement of relative/preferential attention and jointly analyze it with EEG. First, we examined how ASD and TD groups differ in their RALD at two videos displaying complex dynamic social and nonsocial audiovisual stimuli, as well as a neutral, less complex video (bubbles cascading) that did not involve sound. Second, we investigated how RALD correlates with brain activity, as reflected in the *Relative Power Spectral Density* (*RP*) of the EEG signal in four frequency bands. While the joint study of EEG and looking behavior was previously used in EEG artifact correction^38^ and in the studies of human reading^39–42^, to the best of our knowledge only one study made an attempt to jointly analyze looking behavior and EEG in relation to social attention^36^. Our study goes further, proposing to use *RALD* as a measure of preferential attentional behavior and study its association with EEG.

## Methods

### Participants

All caregivers/legal guardians of participants gave written, informed consent, and the study protocol was approved by the Duke University Health System Institutional Review Board. Methods were carried out in accordance with institutional, State, and Federal guidelines and regulations.

#### ASD participants

Participants were 31 children with ASD (23 males, 8 females) between 28 and 81 months of age (mean = 55.3, SD = 14.8). Children with ASD were part of a single site, prospective, randomized, double-blind, parallel group study of placebo versus a single intravenous autologous or allogeneic, unrelated cord blood (CB) infusion in ASD children aged 2–7 years. The trial was conducted under IND #15949. Only data from the baseline visit, which were collected before infusions, were used in this analysis. Clinical diagnosis of ASD was based on the Diagnostic and Statistical Manual of Mental Disorders, Fifth Edition (DSM-5)^43^, and established by expert clinicians using the Autism Diagnostic Observation Scale (ADOS-2)^25^ and the Autism Diagnostic Interview, Revised (ADI-R)^44^. Additional inclusion criteria included (1) stability on current medications for at least 2 months prior to the infusion, (2) participants and parents/guardians were English speaking, and (3) availability of autologous umbilical cord blood unit or ≥4/6 HLA-matched allogeneic unrelated umbilical cord blood unit from the Carolinas Cord Blood Bank. Exclusion criteria included (1) a history of prior cell therapy, (2) use of intravenous immunoglobulin (IVIG) or other anti-inflammatory medications (with the exception of NSAIDs), (3) known genetic syndrome (e.g., Fragile X), presence of dysmorphic features, pathogenic mutation or copy number variation associated with ASD, and/or other significant medical and/or psychiatric comorbidity, (4) obvious physical dysmorphology, (5) an uncontrolled seizure disorder, (6) significantly impaired renal or liver function, (7) known active CNS infection, evidence of uncontrolled infection, and/or HIV positivity, (8) family unwilling or unable to commit to study-related assessments, and/or (9) clinically significant abnormalities in complete blood count. The mean Full Scale IQ of ASD study participants was 80.4 (SD = 21.9) based on the Mullen Scales of Early Learning Composite Score^45^ or Differential Ability Scales Second Edition (DAS-II)^46^.

#### Typically developing participants

Children who did not have a diagnosis or suspected diagnosis of ASD were recruited from the community and the Duke Center for Autism and Brain Development research registry to participate in a study of preschool age children with and without autism. A randomly chosen subset of these children (N = 31) who were age matched to the ASD participants were included in the current analyses. Participants were 31 children (14 males, 17 females) between 39 and 71 months of age (mean = 53.3, SD = 10.5). Children were eligible to be in the TD control group if they had scores on the Strengths and Difficulties Questionnaire (SDQ) that were within the normal range for all scales. The SDQ is a parent-report screening tool for measuring internalizing and externalizing difficulties in children^47–49^. Exclusion criteria for this group included having (1) a biological sibling or parent diagnosed with ASD or developmental delay (DD), (2) a genetic disorder (e.g., Fragile X), (3) vision or hearing problems, (4) a significant motor impairment (e.g., cerebral palsy), (5) chronic or acute medical illness, and (5) a seizure in the last year, a seizure disorder, or being on medication for seizures. The mean Full Scale IQ of TD study participants was 114.3 (SD = 13.5) based on the Mullen Scales of Early Learning Composite Score^45^ or Differential Ability Scales Second Edition (DAS-II)^46^. In order to control for IQ differences between the two groups, IQ was used as a covariate in the analyses.

### EEG measures

#### Protocol

**Figure 1:**
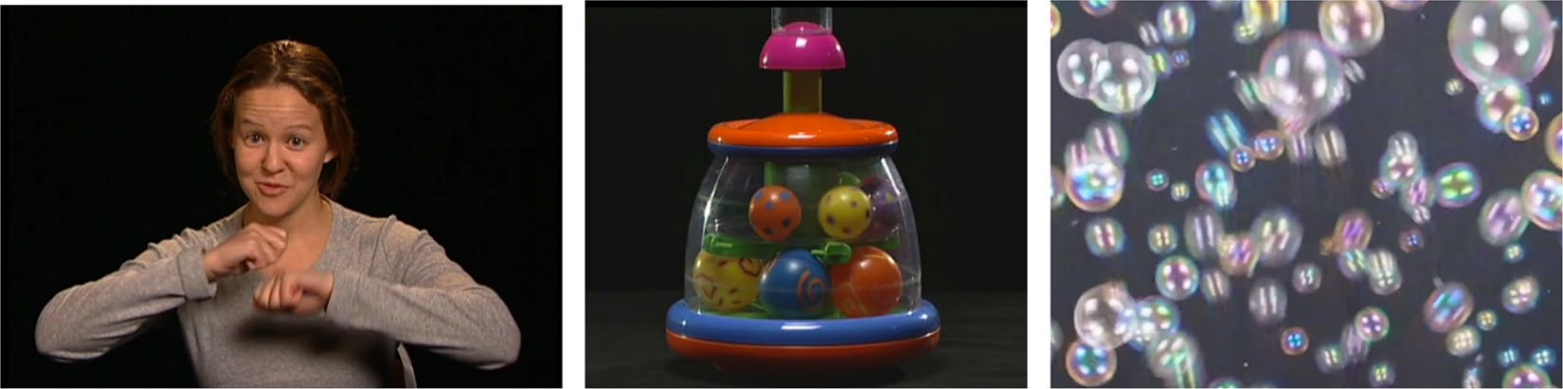
Screenshots of three stimuli used in the study. Left – Social, Center – Toys, Right - Bubbles stimulus.

Continuous EEG was recorded while the participant watched three video stimuli which were each shown twice (total of 6 videos, 6 minutes). Video content was dynamic stimuli consisting of a woman singing nursery rhymes while she gestured (“Social,” video 1), brightly colored dynamic toys that made noise (“Toys,” low social content, video 2), and bubbles cascading across the screen with no auditory content (“Bubbles,” low social and audio content, video 3), see Fig. 1 for corresponding screenshots. The order of Social and Toys videos was counterbalanced to eliminate any potential order effects, and Bubbles was always shown last. During the experiment, two behavioral assistants accompanied the child. They ensured that standard conditions were in place during each experiment, including dimming the lights, seating participants in their parent’s lap in a comfortable armchair 65 inches from the monitor, and redirecting participants in instances of movement and/or poor attention to the videos. The child’s face was recorded from a camera beneath the screen synchronized with the EEG. It allowed post-session editing of periods of inattention. EEG data were recorded from 124 channels with reference to Cz using a Hydrocel Geodesic Sensor Net and Net Amps 400 amplifier (Electrical Geodesics, Eugene, Oregon). Data were collected using Netstation 4.5.6 with a sampling rate of 1000 Hz.

#### EEG preprocessing and data attrition

Data were processed with Matlab 2014a, using the open source Fieldtrip^50^ and EEGLAB^51^ toolboxes for all operations on EEG data. Data were filtered with a 1–100 Hz bandpass filter and a 60 Hz notch filter. Participant videos were inspected for gross inattention to the video and movement artifact, and these time points were removed from EEG analyses. For each participant’s data, persistent bad channels that were deviant in 33% of trials (identified with Fieldtrip function ft_rejectvisual based on within channel variance and kurtosis) were interpolated using spline interpolation, as implemented in the Fieldtrip function ft_repairchannels. Interpolation was chosen to keep consistent datasets across participants. Amount of interpolated channels was between 4 and 21 for ASD participants and 6 and 24 for TD participants. Data were decomposed using Second Order Blind Identification (SOBI) as implemented in EEGLAB^51,52^. Topographic maps of SOBI components were inspected and electrooculogram (EOG) and electromyogram (EMG) components were removed. Forty one-second epochs from each of two presentations of the stimulus with minimal movement contamination were retained (again using ft_rejectvisual function). Data were then re-referenced to the common average as laid out in Nunez and Srinivasan, 2006^53^ using Fieldtrip ft_preprocessing function^50^. These preprocessing methods are commonly employed in modern electroencephalography^50–54^. Finally, a fast Fourier transformation (FFT) was performed on the rectangular windowed time series. For each of the 3 stimulus conditions presented twice, the presentation with the least amount of movement artifact was chosen for analysis.

#### EEG data analysis

As a result of preprocessing step, EEG data were a 3-dimensional array of voltages, with dimensions 40 × 124 × 1000 (40 one-second epochs, 124 channels, 1000 samples per second). Three scalp regions of interest (Regions: Frontal, Central, and Posterior), per McEvoy *et al*.^54^, were used in the analysis. Twelve channels covering the left hemisphere, right hemisphere, and midline were included in each of the three regions^35^. Per each participant and each condition, average Power Spectral Density (PSD) from 40 artifact-free seconds of EEG recording for each channel was binned into four power bands: theta (5–7 Hz), alpha (8–10 Hz), beta 1 (11–20 Hz), and beta 2 (21–30 Hz)^35^. Relative Power Spectral Density (*RP*) was calculated by dividing PSD in each band by the total signal power between 3 and 30 Hz for each channel, resulting in four *RP* values per channel during each video stimulus. *RP* per region was then calculated by averaging values from twelve channels within the region. Additionally, log-ratio of *RP* between pairs of video stimuli V1 and V2 was computed, as well as log-ratio of Theta/Beta power ratio,

$$\begin{array}{rcl}L{R}_{V1-V2,band,ROI} & = & \log (\frac{R{P}_{V1,.band,ROI}}{R{P}_{V2,band,ROI}}),\\ L{R}_{TBR,V1-V2,ROI} & = & \log \,(\frac{R{P}_{V1,theta,ROI}/(R{P}_{V1,beta1,ROI}+R{P}_{V1,beta2,ROI})}{R{P}_{V2,theta,ROI}/(R{P}_{V2,beta1,ROI}+R{P}_{V2,beta2,ROI})}).\end{array}$$

### Measurement of relative average look duration

By comparing within-subject relative average look duration to nonsocial versus social stimuli, each participant’s attention to one type of stimulus served as a “baseline” for comparing that participant’s attention to the second type of stimulus. Attention was coded as a binary signal based off the same video recording used for preprocessing EEG data. The following summary features were extracted from this binary signal:

1. Total looking duration (*TLook*_*V*_) - total amount of time child was watching the screen when video stimulus V was presented;
2. Average look duration (*ALD*_*V*_) - This attention/looking variable was computed as follows for each video stimulus V:
  $$AL{D}_{V}=\frac{TLoo{k}_{V}}{\#Number\,of\,looks\,at\,the\,screen\,during\,the\,video\,presentation}\cdot $$
  With this variable we are providing a measurement of the intermittent behavior of attention.
3. Relative average look duration (*RALD*) – This core new measurement was computed as:

$$RAL{D}_{V1,V2}=\frac{AL{D}_{V1}-AL{D}_{V2}}{AL{D}_{V1}+AL{D}_{V2}}.$$

This measure can be considered as a measure of engagement in one type of video (V1), treating another as a baseline level (V2). As can be seen from the formula, *RALD*_*V1,V2*_ takes values in the range of [−1, 1]. For example, full engagement in Social video and disengagement in Toys video means *RALD*_*Social,Toys*_ = 1, and, vice versa, *RALD*_*Social,Toys*_ = −1 means full engagement in Toys video and disengagement in Social video.

### Statistical analysis methods

To evaluate the ability to distinguish the ASD from the TD group using the attention measures, two-way ANCOVA was performed for *ALD*_*V*_, with categorical predictors of Group (ASD/TD) and video type (Bubbles/Social/Toys), and IQ, Sex, and Age as covariates. Then, one-way ANCOVA was performed for *RALD*_*V1,V2*_ with predictor of Group and the same covariates. In the order of increasing complexity, we applied the following models, each time testing whether a new predictor significantly increased explanatory power of the model:

1a $${\rm{ALD}} \sim {\rm{IQ}}$$

1b $${\rm{ALD}} \sim {\rm{Sex}}+{\rm{IQ}}$$

1c $${\rm{ALD}} \sim {\rm{Age}}+{\rm{Sex}}+{\rm{IQ}}$$

1d $${\rm{ALD}} \sim {\rm{Group}}+{\rm{Age}}+{\rm{Sex}}+{\rm{IQ}}$$

1e $${\rm{ALD}} \sim {\rm{Group}}+{\rm{VideoType}}+{\rm{Age}}+{\rm{Sex}}+{\rm{IQ}}$$

1f $${\rm{ALD}} \sim {\rm{Group}}+{\rm{VideoType}}+{\rm{Group}}:{\rm{VideoType}}+{\rm{Age}}+{\rm{Sex}}+{\rm{IQ}}$$

A univariate linear modeling approach was used to search for possible associations between the proposed baselined attention measures (*RALD*_*Social,V2*_*)* and EEG signal features, namely log-ratio of *RP* (relative power) and TBR (Theta/Beta ratio). In other words, $L{R}_{Social-V2(choiceofnonsocial)}$ and $L{R}_{TBR,Social-V2(choiceofnonsocial)}$ were treated as dependent variables, while *RALD*_*Social,V2*_, Group, and interaction between *RALD*_*V1*__,__*V2*_ and Group were taken as predictors, with Age, Sex, and IQ treated as covariates. This is all expressed in the following equation:

2 $$\begin{array}{ccc}(L{R}_{Social-V2,band,Region,}\,or\,L{R}_{TBR,Social-V2,Region}) & = & {\beta }_{0}+{\beta }_{1}(RAL{D}_{Social,V2})+{\beta }_{2}Group\\  &  & +\,{\beta }_{3}Group\ast (RAL{D}_{Social,V2})\\  &  & +\,{\beta }_{4}Age+{\beta }_{5}Sex+{\beta }_{6}IQ.\end{array}$$

In all models we were interested in the effect of *RALD*_*Social,V2*_ and an interaction term (Group * *RALD*_*Social,V2*_) on the dependent variable. False Discovery Rate (FDR)^55^ correction was applied to p-values corresponding to these 2 regression coefficients from all models of the above type. Initially, FDR correction was done for 60 tests, since both *LR* and *RP*_*Social*_ were used as dependent variables. However, results for *RP*_*Social*_ were not directly interpretable. For the sake of reproducibility, we recomputed FDR for 30 tests. Nothing changed in the significance of the results, only FDR-corrected p-values slightly changed.

For those models that proved significant on RALD or Group*RALD interaction we tested whether adding the interaction term significantly improved explanatory power of the model, by applying 3 models in the order of increasing complexity:

- 3 $$(L{R}_{Social-V2,band,Region,}\,or\,L{R}_{TBR,Social-V2,Region})={\beta }_{0}+{\beta }_{2}Group+{\beta }_{4}Age+{\beta }_{5}Sex+{\beta }_{6}IQ.\,$$
- 4 $$(L{R}_{Social-V2,band,Region,}\,or\,L{R}_{TBR,Social-V2,Region})={\beta }_{0}+{\beta }_{1}(RAL{D}_{Social,V2})+{\beta }_{2}Group+{\beta }_{4}Age+{\beta }_{5}Sex+{\beta }_{6}IQ.$$
- and Model from Eq. (2).

## Results

### Average look duration

**Figure 2:**
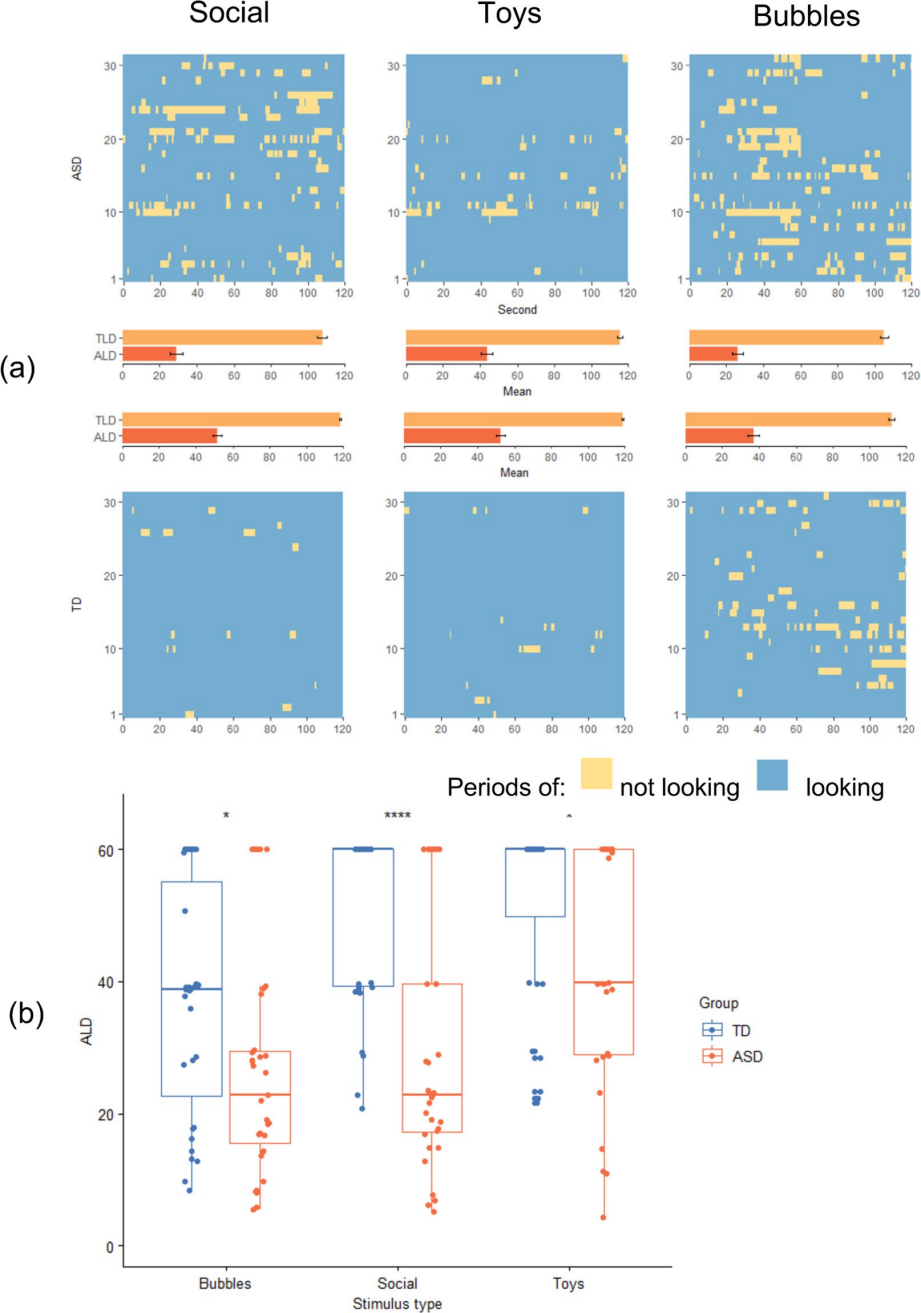
(**a**) Attention measurements in this study. Each line (31 lines total) on each one of the six images represents one participant’s attention during the course of 120 seconds (2 video repetitions of 60 seconds length). (**b**) Behavior of *ALD*_*V*_ for each video type. Asterisks mark level of significance on two-sample t-test between two groups within each video type. *p < 0.05, ****p < 0.0001.

First, and for the sake of completeness, we report initial results on *ALD*, since it forms the basis of *RALD, which is the main subject of this work*. Patterns of attention during each type of stimuli for both groups can be seen on Fig. 2a. Our sequential tests for explanatory power revealed that it increases by adding Group (Eq. 1d), Video type (Eq. 1e) and Group*Video type interaction (Eq. 1f) (p < 0.05, p < 0.001, p < 0.05 on F-tests respectively), even after controlling for differences in IQ. We observed a strong effect of Group, such that ASD children exhibited shorter look durations than the TD group (F_1,177_ > 35.39, p < 0.001), and of video stimulus type, with both groups most engaged in Toys and least engaged in Bubbles (F_2,177_ > 16.04, p < 0.001). Further, an interaction effect was significant (F_2,177_ > 3.21, p < 0.05), suggesting that relative level of engagement between Social, Toys and Bubbles was different in ASD group and TD group; in the ASD group, decreased ALD was most evident while viewing the Social video, which was not the case for the TD group. See Fig. 2b for details. This effect also became evident when considering RALD measures below.

### Relative average look duration

The ability of 3 different $RAL{D}_{V1,V2}$ measures (contrasting Social vs. Toys, Social vs. Bubbles, and Toys vs. Bubbles videos) to distinguish ASD from TD group was explored. To this end, one-way ANCOVA models with Sex, Age and IQ confounding variables were exploited. *RALD*_*Social,Bubbles*_ and *RALD*_*Social,Toys*_ (which can be considered Social vs. Nonsocial) demonstrated significant ability to separate between the groups (F_1,57_ > 4.43, p < 0.04 and F_1,57_ > 10.50, p < 0.002 respectively), while *RALD*_*Toys,Bubbles*_ (two nonsocial stimuli) did not (F_1,57_ > 0.50, p < 0.48).

### EEG measures

We selected *RALD*_*Social,Toys*_ for combined analysis with EEG since it better separated groups (see above), and being a relative measure it eliminated potential effects of baseline mood, excitation, or drowsiness on the day of the experiment. It also follows independent findings about the value of changes/differences (*RALD*) contrary to absolute behaviors^4,21^.

When taking the log-ratio of *RP*_*Social*_ and *RP*_*Toys*_ (which is a measure of relative brain activation while viewing social versus nonsocial stimuli; see Dawson *et al*.^27^), different patterns of associations between *RALD*_*Social,Toys*_ and EEG measures became evident. Specifically, *LR*_*Social-Toys*,*Theta*,*Central*_ and *LR*_*Social-Toys,Theta,Posterior*_ had a positive association with *RALD*_*Social,Toys*_ in the TD group and negative association with *RALD*_*Social,Toys*_ in the ASD group at the level of p < 0.1, while inverse patterns of association were observed in the Beta 1 Frontal band (p < 0.05).

**Figure 3:**
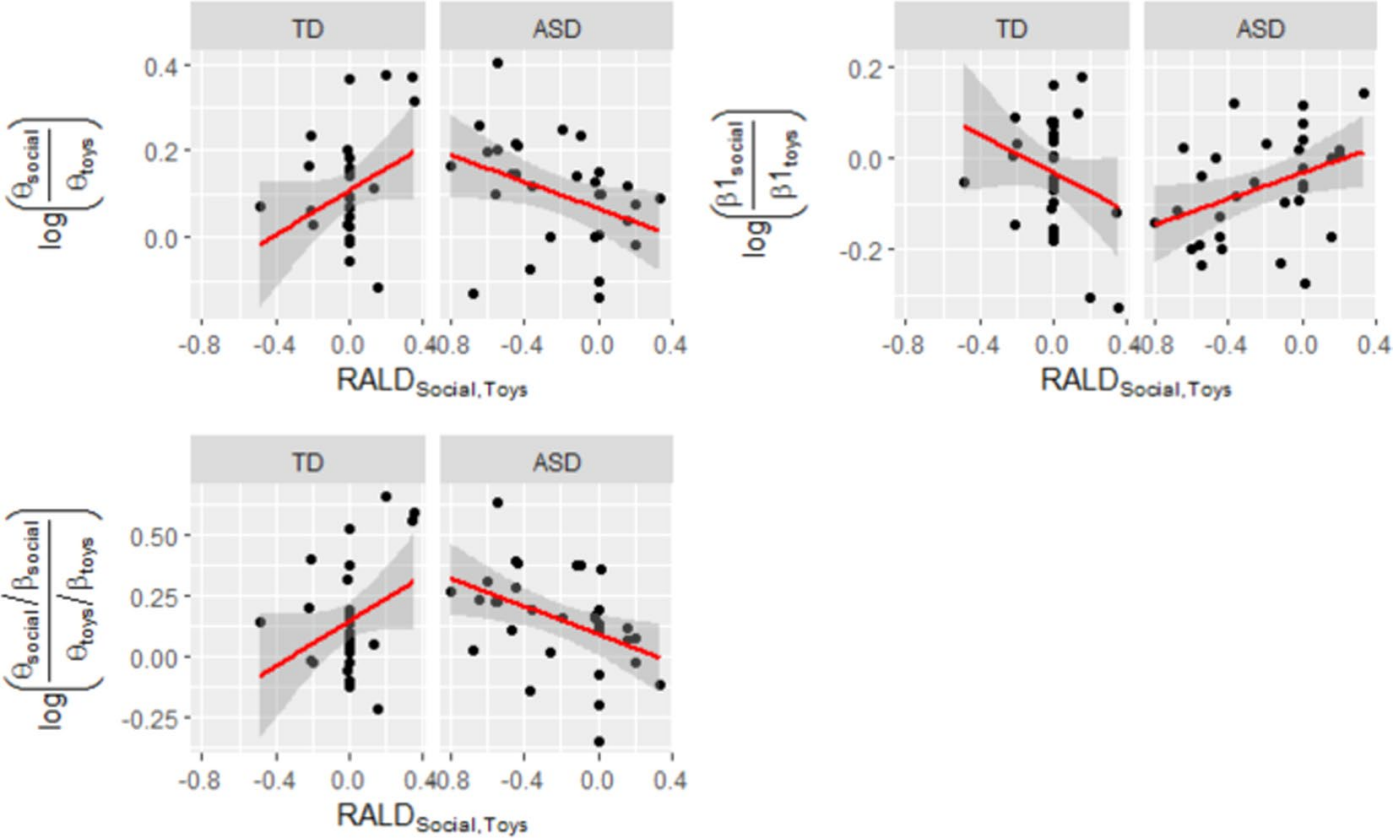
Relations between *RALD*_*Social,Toys*_ and *LR*_*Social-Toys,Theta*_, *LR*_*Social-Toys,Beta 1*_, and *LR*_*TBR,Social-Toys*_ in Posterior Region for TD and ASD groups. Log-Ratio (LR) takes on relative powers of frequency bands as arguments. Results are typical for all the Regions.

The log-ratio of Theta-Beta Ratio (*LR*_*TBR,Social,Toys*_) measure showed significant positive association with *RALD*_*Social,Toys*_ (in TD group, all regions) and significant negative association (in ASD group, Posterior region), or a tendency to negative association (in ASD group, Frontal and Central regions), as can be seen from confidence intervals shown in Fig. 3 and Table 1. Our sequential tests for explanatory power demonstrated that simply adding *RALD*_*Social,Toys*_ to the model containing only covariates and Group (Eq. 4 compared to Eq. 3) did not improve the explanatory power of the model regardless of region and frequency band. However, adding RALD_Social,Toys_ and Group * RALD_Social,Toys_ interaction (Eq. 2 compared to Eq. 3) increased explanatory power in all models that we reported significant above (p < 0.05 on F-test).

**Table 1:** Associations of log-ratio of EEG RP in Social and Toys videos and LR_TBR__,__Social-Toys_ and RALD.

log(RP_Social/_RP_Toys_), Region	RALD_Social,Toys_		Group *RALD_Social,Toys_		Confidence Interval for $\beta$coefficient at RALD_Social,Toys_			
	$\beta$	p-val (FDRcorr)	$\beta$	p-val (FDRcorr)	In TD group		In ASD group	
Theta F	0.2304	0.150	−0.3122	0.097	−0.042	0.502	−0.219	0.056
**Theta C**	0.2950	0.070	**−0.3881**	**0.042**	***0.029***	***0.561***	**−0.228**	**0.041**
**Theta P**	0.2911	0.094	**−0.4389**	**0.041**	***0.008***	***0.574***	**−0.291**	**−0.005**
Alpha F	0.1220	0.527	−0.0027	0.989	−0.232	0.476	−0.060	0.298
Alpha C	0.0108	0.982	0.2416	0.275	−0.328	0.350	0.081	0.424
Alpha P	−0.1715	0.405	0.3648	0.141	−0.537	0.194	0.009	0.378
**Beta1 F**	**−0.3124**	**0.041**	**0.4514**	**0.021**	*−0.545*	**−0.080**	***0.021***	***0.257***
Beta1 C	−0.1401	0.275	0.2130	0.166	−0.371	0.091	−0.044	0.190
**Beta1 P**	−0.2460	0.137	**0.4019**	**0.042**	**−0.520**	**0.028**	***0.017***	***0.295***
Beta2 F	−0.3480	0.204	0.3734	0.228	−0.817	0.121	−0.212	0.263
Beta2 C	−0.2036	0.275	0.1314	0.527	−0.533	0.126	−0.239	0.094
Beta2 P	−0.3355	0.134	0.4758	0.065	−0.701	0.030	−0.045	0.325
**LR**_**TBR**_ **F**	**0.5609**	**0.041**	−**0.7426**	**0.021**	***0.156***	***0.966***	−**0.387**	**0.023**
**LR**_**TBR**_ **C**	**0.4599**	**0.042**	−**0.5829**	**0.041**	***0.093***	***0.826***	−**0.308**	**0.062**
**LR**_**TBR**_ **P**	**0.5824**	**0.042**	−**0.8842**	**0.021**	***0.115***	***1.050***	**−0.538**	**−0.065**

EEG measurements were aggregated to three regions, frontal (F), central (C), and posterior (P). Spectral power was binned into four frequency bands: Theta (5–7 Hz), Alpha (8–10 Hz), Beta 1(11–20 Hz), Beta 2 (21–30 Hz). Associations in bold are significant. In “Confidence Interval” section in case at least one association is significant, for each group positive associations are marked in bold underline with italic, negative associations in bold underline, and no association in bold.

While our primary hypothesis involved comparing brain activity across two audiovisual conditions that differed in social versus nonsocial content, we also carried out similar analyses comparing the social and bubbles conditions and the toys and bubbles conditions. In this case, the two conditions differed not only in content but also level of stimulation because the bubbles condition did not involve audio. No significant results were found.

## Discussion

In order to illustrate the value of jointly studying attentional behavior and EEG, we first investigated a metric for average look duration (*ALD*), defined as the average length of separate looking periods (intermittent attention) to a complex, dynamic stimulus. We found that, compared to age matched TD children, children with ASD have shorter average look durations to both social and nonsocial complex dynamic audiovisual stimuli. ALD is the building block for a newly proposed measure of *relative* average look duration (*RALD*) to different stimulus types; *RALD* to the social compared to nonsocial stimuli exhibited differential associations with neurophysiological measures for the ASD and TD groups. These results indicate that the neural systems that mediate relative differences in sustained attention to social versus nonsocial stimuli are not the same for children with ASD versus TD. Additionally, adding an interaction term, thus accounting for differential associations in TD and ASD, significantly increased explanatory power of the model even after controlling for group differences in IQ and sex. These results therefore support the idea that ASD and TD children process social and nonsocial stimuli differently and that combining simultaneously recorded attentional behavioral data and EEG data adds explanatory value in understanding these differences.

Group differences in *ALD* were most robust when the children were viewing the social stimulus, further supporting the use of *RALD* to capture differential attention between different stimulus types. That is, ASD children had shorter look duration for all stimuli types, but the effect was most pronounced in the social condition. When the relative measure (RALD) was examined, it was found that the contrast between social and nonsocial stimuli (*RALD*_*Social,Toys*_, *RALD*_*Social,Bubbles*_) distinguished the TD and ASD groups, while the contrast between two nonsocial stimuli (*RALD*_*Toys,Bubbles*_) did not yield group differences. ASD deficits in sustained attention appear to be strongest when social content is involved, highlighting a context-specific difference in attention. This may distinguish ASD from other disorders of attention, including attention-deficit/hyperactivity disorder (ADHD) and schizophrenia. Our findings are consistent with other studies that have shown differences between ASD and TD children in total looking time and peak look duration in the context of social attention^6,12,14,15,24^. However, these studies are limited in that they did not measure average look duration and only focused on maximum and total durations.

As the contrast between the Social and Toys stimuli for average look duration (*RALD*_*Social,Toys*_) was most robust, we used it for subsequent investigation of its relationship with simultaneously recorded EEG, where the measure of interest was relative EEG power during the social as compared to nonsocial stimuli. Analysis revealed that correlations between RALD for social versus toys stimuli and underlying patterns of EEG differed for children with ASD versus TD. For TD children, as average look duration to social stimuli relative to toys increased, central and posterior EEG theta power while viewing social versus toy stimuli also increased. This is consistent with previous studies that have found that frontal theta band activity increases when individuals pay attention to multi-sensory stimuli involving auditory and visual input^56^, particularly since the social stimuli were more complex (e.g., involving language) than the dynamic toy stimuli. Furthermore, reduced frontal beta power while viewing social versus toy stimuli was associated with increased attention duration to the social relative to the nonsocial video. Studies have shown that working memory encoding is associated with a transient reduction in beta power (see review by Hanslmayr, Matuschek^57^). The social video involves the actress speaking and gesturing to the child which might have invoked working memory processes.

Different patterns emerged in the ASD group. Increased posterior theta power and decreased frontal and posterior beta power while viewing the social versus toy stimuli was associated with shorter average look durations to the social video relative to the nonsocial video. Taken together, we conclude that TD children with preferential attention to social vs nonsocial stimuli exhibit an expected brain response while watching the social stimuli that is characterized by high levels of theta power and low levels of beta power across the scalp. ASD children, however, appear to show the opposite effect or no association at all. This suggests that even when ASD children show preferential attentional engagement with social content, their underlying brain activity is not the same as the TD children. Given the relatively small sample size and the fact that we did not make a priori predictions regarding these associations for the ASD group, replication with a larger sample size is needed.

Our findings, showing main differences between ASD and TD children in the associations of looking behavior and EEG in theta and beta bands, prompted us to study a metric, typically used in ADHD research, Theta-Beta ratio (TBR)^58–60^, which measures an increase in theta power relative to a decrease in beta power. Indeed, TBR log-ratio was positively associated with *RALD*_*Social,Toys*_ for TD children across all scalp regions, while a negative association existed for ASD children across the posterior region. There has been evidence for use of TBR as a biomarker for ADHD, but the exact neural basis of the TBR is still poorly understood (see Lenartowitz, Loo^58^ and Jeste *et al*.^61^ for reviews). However, no previous research to our knowledge has looked into TBR as a measure of brain response to social/nonsocial stimuli, especially in children with autism. Interestingly, it is estimated that 37–85% of the ASD population has comorbid ADHD^62^. While both disorders involve disruptions in attention, individuals with ADHD show more pronounced deficits in sustained attention than those with ASD^63,64^. Since TBR is atypical in both ADHD and ASD, we may be probing attentional brain circuitry that is commonly disrupted in both disorders. More work is needed to understand the similarities and differences in brain functioning and attentional behaviors between ASD and ADHD participants.

Previous research has studied EEG activity during social and nonsocial videos in clinical trials for children with ASD. In a study comparing a TD group and two behavioral intervention models (Early Start Denver Model^9^ and community intervention), Dawson *et al*.^27^ reported increased log-ratio (Faces vs. Objects) theta power in the TD group as well as the ESDM group, while the opposite pattern was observed in the group that received community intervention. In the present study we found that, for the TD group, increased theta power during the social stimulus and log-ratio (Social vs. Toys) was associated with increased preference to social videos, consistent with Dawson’s findings in the TD and ESDM groups. Increases in theta power have been implicated in the allocation of greater attentional and cognitive resources^27^. Furthermore, Murias *et al*.^35^ found that higher baseline beta power was predictive of changes in the Vineland Socialization subscale score in an open-label trial testing the efficacy of umbilical cord blood for children with ASD. It is clear from these studies that theta and beta power are viable, modifiable biomarkers for ASD, and the current results provide additional evidence that these brain markers are associated with the ability to sustain attention, which involves development of inhibitory and executive functioning skills. Data shows that there is similar association in ASD children in our study (log-ratio of beta power increases as engagement in social stimulus increases), while this pattern is not showing up in TD children. This supports the idea that ASD and TD children process social and nonsocial stimuli differently, and combining behavioral and EEG data is a way to reveal this difference.

A potential weakness in our study was the fact that TD and ASD groups differed not only in terms of an ASD diagnosis but also in cognitive ability and sex distribution, with the ASD group having a mean lower IQ and more male participants compared to the TD group. All results accounted for this group difference by including IQ and sex as covariates. However, future work should include comparisons between groups with similar cognitive ability and sex distribution. In addition, the study used a relatively small sample size which limited statistical power; nevertheless, many findings were still robust enough to be statistically significant.

In the presented work we proposed a method to analyze looking behavior during synchronized spontaneous EEG recording. We showed that the measures of looking behavior that incorporate not only total attention time but also average look duration, differentiate between children with TD and ASD and are associated with differential patterns of EEG activity, which differ in children with ASD and TD. Future work will aim at combining this measure with EEG signal features for improving assessment of autism spectrum disorder.

## Data Availability

Groups interested in direct use of the data can do so via collaboration with the authors due to privacy and consent considerations. Data is stored in a secured partition at Duke Health.
